# Automatic Detection of Cervical Cancer Cells by a Two-Level Cascade Classification System

**DOI:** 10.1155/2016/9535027

**Published:** 2016-05-19

**Authors:** Jie Su, Xuan Xu, Yongjun He, Jinming Song

**Affiliations:** ^1^College of Computer School, Harbin University of Science and Technology, Harbin 150080, China; ^2^Department of Pathology and Lab Medicines, H. Lee Moffitt Cancer Center and Research Institute, Tampa, FL 33612, USA

## Abstract

We proposed a method for automatic detection of cervical cancer cells in images captured from thin liquid based cytology slides. We selected 20,000 cells in images derived from 120 different thin liquid based cytology slides, which include 5000 epithelial cells (normal 2500, abnormal 2500), lymphoid cells, neutrophils, and junk cells. We first proposed 28 features, including 20 morphologic features and 8 texture features, based on the characteristics of each cell type. We then used a two-level cascade integration system of two classifiers to classify the cervical cells into normal and abnormal epithelial cells. The results showed that the recognition rates for abnormal cervical epithelial cells were 92.7% and 93.2%, respectively, when C4.5 classifier or LR (LR: logical regression) classifier was used individually; while the recognition rate was significantly higher (95.642%) when our two-level cascade integrated classifier system was used. The false negative rate and false positive rate (both 1.44%) of the proposed automatic two-level cascade classification system are also much lower than those of traditional Pap smear review.

## 1. Introduction

According to the statistics of WHO (World Health Organization), there were 530,000 new cases in the world in 2012 and it caused the second highest mortality rate in cancers of female patients. More than 270,000 females died from cervical cancer every year in the world, more than 85% of which occurred in the developing countries [1]. The screening of cervical cancers in the developing countries encountered serious difficulties, due to backward economy and poor condition. The incidence of cervical cancer is 6 times higher in the developing countries than in developed countries. Therefore, there is an urgent need to develop a screening method that is appropriate for the developing countries.

Cervical cancer is typically diagnosed by the liquid based cytology (LBC) slides followed by pathologist review. This method overcomes the problem of fuzzy background, cell overlap, and uneven staining of traditional methods and improves the sensitivity of screening [2]. However, the human review of the slides carries the price of large screening volume, high cost, and dependence of the reliability and accuracy on the reviewers' skill and experience. These factors reduced the accuracy of the screening method and resulted in relatively high false positive (~10%) or false negative rates (~20%) [3].

Automatic and semiautomatic methods have been used to identify abnormal cells from the slides by analyzing the contours of the cells [4–9]. Automatic analysis method of cervical cell images has recently been developed and is used to detect cervical cancers and has been intensively studied and improved. In this method, the cells are smeared on the slides, from which images were obtained by cameras of industrial quality. The images are then analyzed to look for abnormal cells. This method has the benefit of saving huge resources of mankind and materials and greatly improved the efficiency of screening, reduced human errors, and enhanced the accuracy of the screening. The acquirement of cell features, design of cell classification system, and the classification of the cells play critical roles in this method. In this study, these three important aspects were investigated.

Different classification systems of cervical smear cells have recently been proposed [6, 10–13]. Chen et al. [6] proposed classifying the cells into superficial cells, intermediate cells, parabasal cells, low-grade squamous intraepithelial lesion, and high-grade squamous intraepithelial lesion (HSIL). Rahmadwati et al. [10, 11] classified all the cervical cells into normal, premalignant, and malignant categories. In another study [11], the premalignant stage was further divided into CIN1 (carcinoma in situ 1), CIN2, and CIN3. Rajesh Kumar et al. [12] classified the cervical cells into two types of cells, normal and abnormal cervical cells. Sarwar et al. [13] divided the cells into three normal cells (superficial squamous epithelial, intermediate squamous epithelial, and columnar epithelial), and four abnormal cells (mild squamous nonkeratinizing dysplasia, moderate squamous nonkeratinizing dysplasia, severe squamous nonkeratinizing dysplasia, and moderate squamous cell carcinoma in situ). These classification systems are still in the stage of research. No system has been finalized as the method for clinical practice. Since the Pap smears are usually contaminated by blood and lymphoid tissues, the method of directly classifying the squamous cells into normal and abnormal cells is not appropriate for the classification of cervical smears.

In regard to the acquirement of cell features, most of the researchers used multidimensional features to classify the cells [12, 14–16]. Some authors analyzed four parameters: area, integrated optical density (IOD), eccentricity, and Fourier coefficients [12]. Other authors used 16 features: area of nucleus, area of cytoplasm, nuclear gray level, cytoplasm's gray level, and so forth [14]. Some authors acquired nine parameters: mean intensity, variance, number of concave points, area, area ratio, perimeter, roundness, entropy, and intensity ratio [15]. Finally, some other authors used 27 parameters, which included contrast, energy, correlation, and homogeneity [16]. Most of these parameters were obtained through computers. It remains to be studied which parameters are more appropriate for cell classification.

In the recent years, the method of multiclassifier integration is the focus of the study for pattern recognition and has been applied to some areas of biological feature recognition [17–19]. In one study [18], neural network classification system based on two-level cascade system has been used to recognize lung cancer cells and has decreased the error rate of single classifier network. In another study [19], two-dimensional cascaded AdaBoost framework was used for eye localization. From these pioneer studies, it is known that the method of classifier integration could help to enhance the sensitivity in comparison to a traditional single classifier.

We hereby proposed the following novel ideas for automatic analysis method of cervical cell images: (1) Feature extraction: obtaining 28 dimensional features including nuclear area and contrast, 20 morphologic, and 8 textural features, based on the difference in cell morphology or cell density. (2) Cell classification: first separating the cells into epithelial cells, lymphoid cells, neutrophils, and junk cell and then classifying the epithelial cells into normal and abnormal cells. (3) Method of classification: classification of cervical cells by two-level cascade. The cells are first classified by fast and rough C4.5 classifier and then by more accurate LR classifier into normal and abnormal cells.

## 2. The Methods

### 2.1. The Workflow of the Automatic Detection System

**Figure 1:**
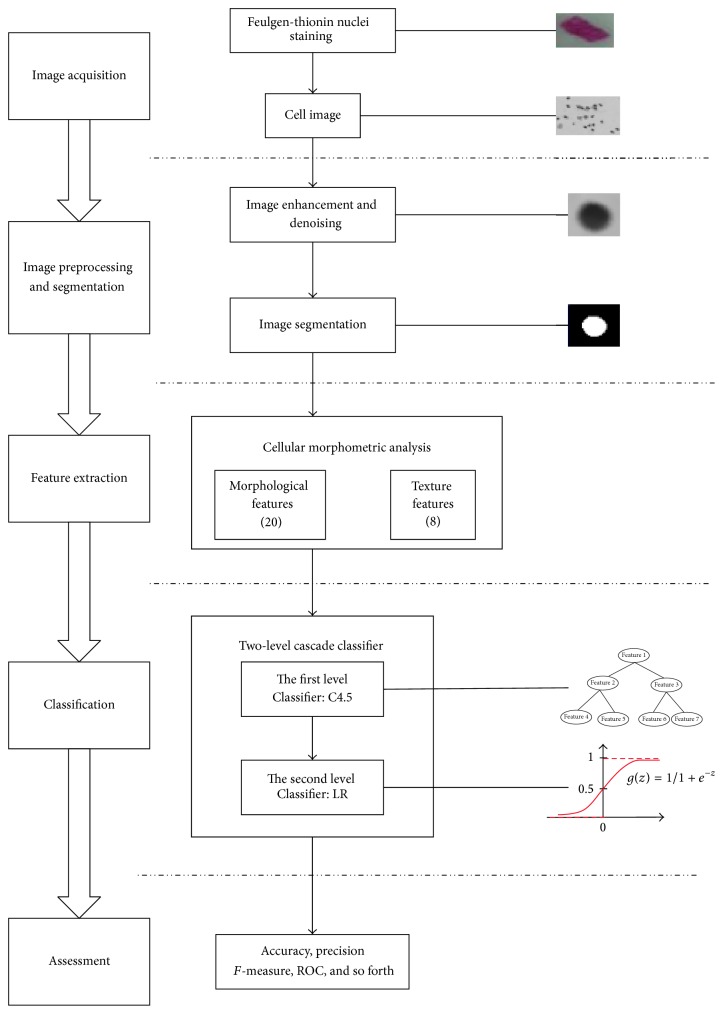
Flowchart of the Automatic Detection System.

The main components of automatic analysis method of cervical cell images include high quality digital camera, computer, three-dimensional moving stage, microscope, and the auxiliary cell analysis equipment. The workflow is shown as Figure 1. The first level classification or rough classification includes acquirement of cell images, image preprocessing, cell nuclei and background segmentation by appropriate cutoff, measurement and analysis of cell morphology, and optical density and textural features, by the cell assessment and classifying function of C4.5. The second level classification or detailed classification includes extraction of similar features as in Step 1 and usage of LR classifier to classify the cells into normal and abnormal epithelial cells.

### 2.2. Acquirement of Cell Images

**Figure 2:**
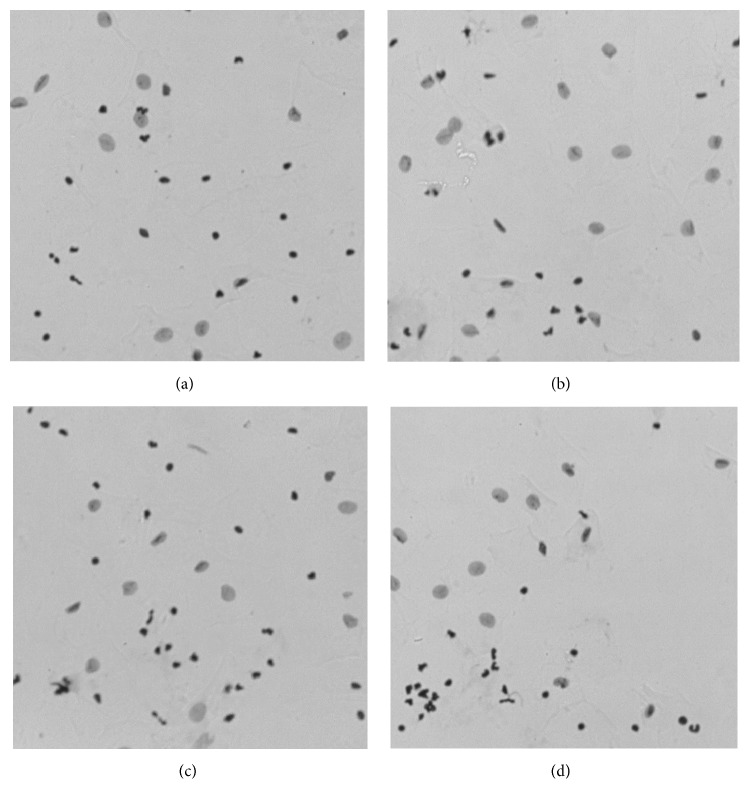
The nuclei after thin liquid based cytology. (a) Sample image 1. (b) Sample image 2. (c) Sample image 3. (d) Sample image 4.

Pap smear has served important functions in cervical cancer screening for the past half century. But it has high requirements on the cell smear and has high false positive rate [20]. In contrast, the technology of liquid based cytology cell analysis showed higher smear quality and sensitivity [21] and reduced the false positive rate and is the most common method of smear preparation. The cervical cancer development is usually accompanied by the nuclear abnormality of the cells. Therefore, we used Feulgen-thionin staining method to make thin liquid based cell smear as shown in Figure 2 and then analyze the smears using automatic analysis method of cervical cell images.

Automatic detection method of cervical cell images acquired cell image though high quality camera (IDS UI-3370CP-C-HQ) on the microscope (Olympus BX43) and saved them as of a 300*∗*300 resolution images in  .jpg format with 8-bit gray depth, then divided the image into 332 areas with each area containing 200–400 cells, and separated and analyzed the cells in each area. Based on the opinions of pathologists and cytotechnologists, we then selected 20,000 cells in images derived from 120 different thin liquid based cytology slides, which included epithelial cells (normal 2500, abnormal 2500), lymphoid cells, neutrophils, and junk cells.

### 2.3. Image Preprocessing and Segmentation

Due to various limitations and interference, there is always reduction in quality during smear making, image acquirement, image transfer, and conversion, such as uneven staining, uneven lightness, noise during transfer, and loss of original quality during conversion. Therefore, it is necessary to do some preprocessing of the images before cell segmentation to assure the accuracy of the analysis [22, 23].

We studied the image enhancement and the noise reduction in cervical cell image analysis. To enhance the image, we stored some of the details of the images before histogram equalization and then restored the details while doing histogram equalization. This method increased the contrast between the cells and the background, made the cell borders stand out, and enhanced the local contrast of the image by removing or reducing the nonrelevant information [4, 24]. To reduce the noise, the median filter method [25, 26] was used in the study to first rank the pixels in the areas according to the degree of grayness from low to high and then select the median value as the output pixel. This was a nonlinear smoothing filtering technology, which could not only inhibit pulse pepper and salt noise, but also protect the image border well.

Cell images segmentation is an important step in the automatic cell recognition. It determines if the cell nuclear morphology will match the original cells and will influence the accuracy of the feature parameters and the sensitivity of the system. The adaptive threshold segmentation method used in this study is a simple but effective method. It determined the best segmentation cutoff by the features and distribution of the peak and bottom of the histogram equalization curve, to separate the nuclei from the background [27, 28]. This method is not only simple and fast in calculation, but also effective in dealing with images with big difference in local background grayness in the targeted areas.

### 2.4. Extraction of Features

The selection of cell features and calculation are the essential steps in cell recognition. There is always contamination of the cervical specimens by lymphoid cells, blood, and junk cells. Based on literature suggestions and different features in cell nuclei, we select 28 features, including 20 morphologic features and 8 texture features.

#### 2.4.1. Morphologic Features

Morphologic features were used to describe the shape of the nucleus, which was important to distinguish between different cell types. According to the different morphology of the nucleus, we extracted 28 morphologic features, including area [12, 15, 29], Circularity, Distance, Sigma, Sides, Roundness, Convexity [29], *I*_*a*_ (centroid coordinates of *x*-axis), *I*_*b*_ (centroid coordinates of *y*-axis), *M*_11_, *M*_02_, *M*_20_, Compactness [29], Count-Length [30], Diameter [30], Radius [29, 30], Rectangularity [30], Anisometry [31], Bulkiness [32], and Structure-Factor [33].

After the cells become malignant, the number of chromosomes in the nucleus will change, leading to changes in the shape of the nucleus. As a result, the nuclear circularity and the roundness will also change. The roundness examines the distance between the border and the center of the area, which can be expressed as

(1) $$\begin{array}{c} \text{Roundness}=1-\frac{\text{Sigma}}{\text{Distance}}, \end{array}$$

where Sigma indicates the deviation from the mean distance and Distance indicates the mean distance, which can be defined as

(2) Sigma=∑x,yg0−gx,y−Distance2Area,

(3) Distance=∑x,yg0−gx,yArea.

*g*_0_ is the mean values of pixel in the area of cell and *g*_(*x*, *y*)_ is the pixel value of dot (*x*, *y*) in the area of cell.

Since the calculation of roundness is associated with the number of sides of equilateral polygons, the number of sides can be used to estimate the roundness roughly. The more sides the equilateral polygon has, the rounder the nucleus is. Sides are defined as

(4) $$\begin{array}{c} \text{Sides}=1.4111*{\left(\;\frac{\text{Distance}}{\text{Sigma}}\;\right)}^{0.4724}. \end{array}$$

Circularity is used to calculate the similarity of the nuclear region with a circle and can be expressed as (5), where *P* is the perimeter:

(5) $$\begin{array}{c} \text{Circularity}=\frac{P^{2}}{4\pi \cdot Area}. \end{array}$$

With the transformation of the shape, size, and location of the image, centroid coordinates will change. Centroid coordinates (*I*_*a*_, *I*_*b*_) can be used to estimate the geometric transformation of the images, which can be expressed as

(6) Ia=h+h2−M20·M02+M112,Ib=h−h2−M20·M02+M112.

*M*_20_ and *M*_02_ separately represent the sum of the pixel values of *x*- and *y*-axis of the nucleus. *M*_11_ represents the mean values of every pixel in the nucleus. *h* was defined in

(7) $$\begin{array}{c} h=M_{20}+\frac{M_{02}}{2}. \end{array}$$

#### 2.4.2. Texture Features

Textural features are the repeated textural units and their patterns of presence. The extraction of textual features is to convert the three-dimensional difference of random texture or geometric texture into the differences in the values of grayness and then use mathematical models to describe the texture information. Symbiotic matrix is an important method in the analysis of the texture features of the images. It represents the combined information of direction, distance, the degree, and the speed of the change of the images, by calculating the relationship of the distance and direction between two points of grayness in the image.

There are 14 dimensional features in the symbiotic matrix proposed by Haralick et al. [34]. The features used by us include Contrast [16, 30], Energy [16, 30], Correlation [16, 30], Homogeneity [16], Entropy [15, 30], Anisotropy [35], Mean, and Deviation.

Mean is used to represent the mean gray values of the nucleus, which can be defined by

(8) $$\begin{array}{c} Mean=\frac{\sum_{x,y}g\left(x,y\right)}{\text{Num}}. \end{array}$$

*g*(*x*, *y*) is the gray values of the pixel (*x*, *y*). Num denotes the number of pixels of images.

The deviation is used to represent the deviation of gray values of the nucleus, which can be represented by

(9) $$\begin{array}{c} \text{Deviation}=\sqrt{\frac{\sum_{x,y}{\left(g\left(x,y\right)-\text{Mean}\right)}^{2}}{\text{Num}}}. \end{array}$$

### 2.5. The Two-Level Cascade Classifier Integration

The false classifications produced by different classifiers usually do not overlap when you analyze the multidimension features of the same entity. It indicates that different classifiers might be complementary in information and might be used to enhance the capability of detection. Single classifier is prone to make incorrect classification or miss the classification, resulting in reduced accuracy of the classification. On the other hand, combing different classifier can achieve higher capability in classification by making using of their complementary information.

**Figure 3:**
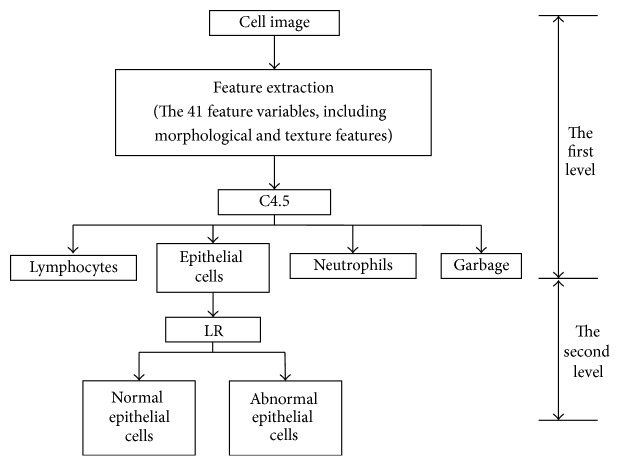
Flowchart of the two-level classification system.

The integration method of combing multiple classifiers can be cascade or parallel. In the cascade method, the output of the previous classifier is used as the input of the next classifier, with each classifier having its own model of feature extraction and model of classification. After analyzing the C4.5 classifier and the LR classifier, we used two-level cascade integration system, that is, another step of classification on the cells that are difficult to classify by C4.5 classifier. In this way, we achieved higher sensitivity of recognition. The flowchart is shown in Figure 3.

The level 1 classification is rough classification and can use fast classifier such as C4.5 to classify the cervical cells into epithelial cells, lymphoid cells, neutrophils, or junk cells. In level 2 classification, the epithelial cells from level 1 classification are further classified into normal and abnormal cells by logic regression classifier with high accuracy.

Every child classifier will give its accuracy, but the final rejection rate is determined by the last output. Therefore, the accuracy and the false rate of each child are independent and do not affect each other. The accuracy and the false rate of the whole system are shown in

(10) C=∑i=12ci,

(11) W=∑i=12wi.

In these equations, *c*_*i*_  and  *w*_*i*_ separately represent the rate of correct recognition and rate of incorrect recognition. Therefore, the function of the integrated system is dependent on the capability of every step, especially the rate of incorrect recognition of the first few steps. In ideal situation, the rate of incorrect recognition of every step is 0. Even if the rate of correct recognition of every step is not high, the final rate of correct recognition will approach 1, as the steps increase.

### 2.6. Assessment Functions

The classifications are evaluated with four parameters. Their accuracy is shown in (12), precision rate in (13), recall rate in (14), and *F*-measure in (15):

(12) accuracy=TP+TNTP+TN+FP+FN,

(13) precision=TPTP+FP,

(14) recall=TPTP+FN,

(15) F-measure=α2+1∗precision∗recallα2precision+recall.

In these equations, TN represents true negatives, TP true positions, FN false negatives, and FP false positive. *F* is the assessment parameter when *α* = 1 in (15).

## 3. The Results

In this study, we selected 20,000 cells, including 2500 abnormal epithelial cells, from 120 different thin liquid based cytology slides based on the cell features and the opinions of the pathologists. During the procedure of cervical specimen collection, not only epithelial cells, but also lymphoid cells, blood, and other contaminants were also collected, which results in reduced recognition rate of these cells. Therefore, direct classification of these five groups of cells is not very helpful to identify the abnormal epithelial cells. So we separated the experiment into two steps to identify the abnormal epithelial cells. In the first step, the cells were separated into epithelial cells, lymphoid cells, neutrophils, and junk cells. In Step 2, we separated the epithelial cells into normal and abnormal epithelial cells.

### 3.1. Image Preprocessing and Segmentation

**Figure 4:**
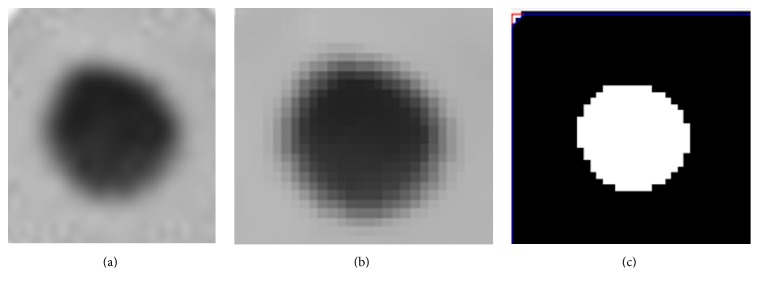
The results of image preprocessing and segmentation. (a) The original image. (b) The image after preprocessing. (c) The image after segmentation.

The purpose of image preprocessing is to reduce the complexity of the image data and to prepare for the analysis in the following steps. To reduce the influence of interference to the image analysis, we used the method of histogram equalization to enhance the image and median value filtering to remove the noise. The results are shown in Figure 4(b). The image segmentation is the process to isolate the image into characteristic areas and select the areas we need for analysis. This step would directly affect the feasibility and the reliability of the next steps. We used the method of adaptive threshold for image segmentation, the results of which are shown in Figure 4(c).

As can be seen in Figure 4, the method we used can effectively reduce the noise and retain the borders of the image. It also shows that the method of segmentation can effectively select out the target areas.

### 3.2. Feature Extraction

The relevant cell features extracted after image segmentation provide the information for automatic image analysis. Therefore, the extracted features should adequately reflect the differences of normal and abnormal epithelial cells. In this study, we extracted 28 morphologic and texture features, which are shown in Table 1.

### 3.3. The Classification of the Cells

In the process of specimen collection, epithelial cells, lymphoid cells, blood, and other contaminants were also collected. It is difficult to achieve satisfactory classification if a single classifier is used. The combination of multiple classifiers can overcome the weakness of a single classifier, make full use of each classifier's strength, and reduce the recognition error rate and increase the recognition robustness [14, 36, 37]. The following is the workflow of the two-level cascade integration system.

**Step 1 (rough classification).**

**Figure 5:**
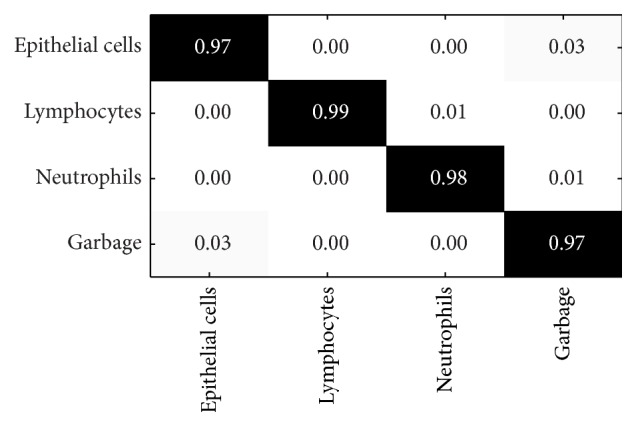
The confusion matrix of the percentages of correct recognition. *x*-axis represents the predicted cell types. *y*-axis is correct cell types. The values in black are the percentages of correct recognition. The values in grey are the percentages of incorrect recognition.

In Step 1 classification, or rough classification, we separated the cells into four groups of cells, which are epithelial cells, lymphoid cells, neutrophils, and junk cells. We used the parameters best for classification to build C4.5 classifier. Confusion matrix shows the relationship between the predicted cell type and the original cell type and further calculates the error rate and precision. The values in normalization confusion matrix represent the percentages. We used C4.5 classifier in the Step 1 classification. The assessment results on the precision of the classification using confusion matrix are shown in Figure 5.

As can be seen in Figure 5, the lymphoid cells are easy to recognize because of the stability of their nuclei. The stability of the neutrophils is less, so its recognition rate was in the middle. The junk cells in the image are contaminants in the specimen collection process, the debris from smear making process, or cell overlap and showed low nuclear stability and therefore had low recognition rate. Epithelial cells including normal cells and abnormal epithelial cells had less nuclear stability and also lower recognition rate.

Even though confusion matrix can indicate the correct recognition rate of every type of cells, it cannot directly tell the overall correctness, precision, recall, *F*-measures, and so forth.  Table 2 shows the results of Step 1 classification. Naïve Bayesian (NB) classifier built from the best parameters is used as the reference group.

As can be seen in Table 2, C4.5 classification is better than NB classifier in accuracy, precision, recall, and *F*-measure. This also indicates that our method of fist classifying the cells into 4 groups is more appropriate for cervical cells. The precision, recall, and *F*-measure of C4.5 are around 96%, while the precision and recall of NB classifier are worse. There is also influence on the *F*-measure.

**Step 2 (epithelial cell classification).**

In this step, we further classified the cells from Step 1 into normal and abnormal epithelial cells. We built LR classifier by using the optimal parameters for classification and compared it with the NB classifier using the same parameters. The overall true positive rate of abnormal epithelial cells is shown in Table 3.

As can be seen in Table 3, LR classifier is much better than NB classifier in both accuracy of the two types of cells (normal and abnormal epithelial cells) and the precision of abnormal epithelial cells. This also indicates that the level 2 classification by LR classifier is more appropriate for cervical cell recognition.

**Figure 6:**
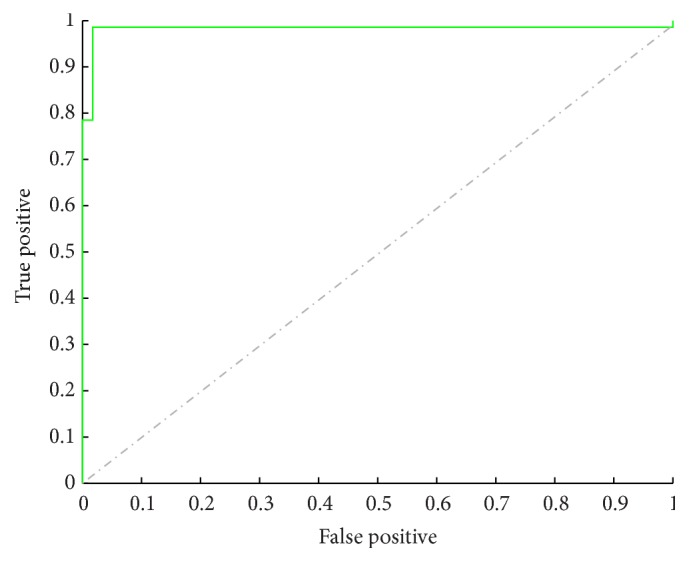
The ROC curves of the results of Step 2 classification. *x*-axis is the false positive. *y*-axis is true positive. The dash and dot line indicate the chance curve. The ROC curve of the results from Step 2 classification is in green color and on the left upper corner.

The assessment of the effectiveness of classifier is dependent not only on overall correctness, but also on specificity and sensitivity. The ROC curve and the areas under the ROC curve in this study not only combine sensitivity and specificity but also take all critical values into consideration and therefore can better represent the correctness of the method used in this study. The ROC curves are shown in Figure 6.

As can be seen in Figure 6, the ROC curve of LR classifier is on the left upper corner and far from the chance curve. The area under ROC curve is 0.996, far bigger than the areas (0.5) under the chance line. The results show significant difference if we adopt the method of classifying the cells into 5 groups by LR classifier and compare it with the chance line without any value.

### 3.4. Further Analysis of the Experimental Results

The final goal of this study is to recognize abnormal epithelial cells and to filter out the interfering lymphoid cells, neutrophil, and junk cells. We used two-step cascade classification system to increase the sensitivity and the specificity of abnormal epithelial cells. To confirm the effectiveness of this cascade method and to show that it is not an effective strategy to directly separate the cells into 5 groups, we used the single classifier system that is commonly used by Weka, the same experimental data, and the same parameters to separate the cells into five groups and then compare with the results produced by our method.

**Figure 7:**
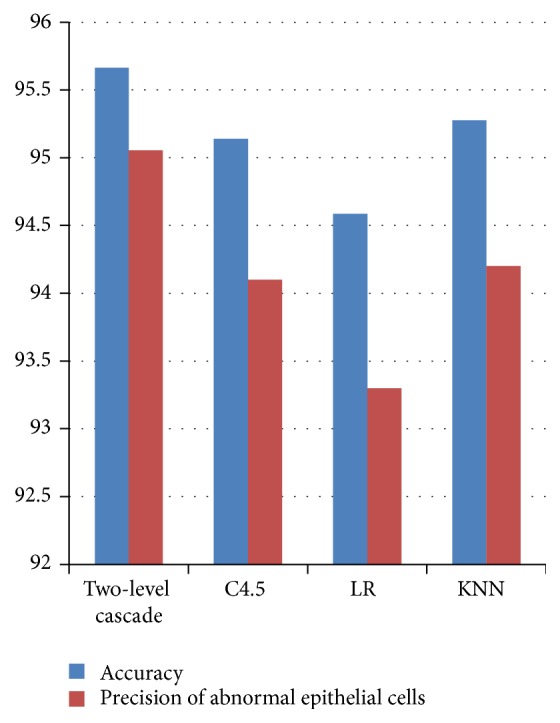
The histogram of the detection accuracy and the precision of each classifying system (KNN: *K*-nearest neighbor).

According to the data from Steps 1 and 2 and (9) and (10), the overall accuracy and precision of detection of abnormal cells of our two-level cascade classification system are 95.805% and 95.642%, respectively. As can be seen in Figure 7, they are higher than the accuracy and the precision of any single classifier system. The detection rate of abnormal cells is also 2% higher than any single classifier system. These results indicate that the two-step cascade classifier integration system not only is appropriate for cervical cancer screening, but also enhances the sensitivity of abnormal epithelial cell detection.

In comparison to the false negative (20%) and false positive (10%) of traditional Pap smear review [3], both the false negative rate and false positive rate (both 36 out of 2500 and 1.44%) of the proposed automatic two-level cascade classification system are much lower. These results demonstrated that the proposed classification system has the potential to dramatically reduce the false negative and false negative rate, to avoid misdiagnosis, and to increase the accuracy and reliability of cervical cancer screening.

## 4. Conclusion

In conclusion, we developed our two-level cascade classifier based on the experience of other studies and obtained 28 dimensional features in morphology and texture. We first separated the cells into epithelial cells, lymphoid cells, neutrophils, and junks cells because of mixed cell types in the specimen and then further classified the recognized epithelial cells into normal and abnormal epithelial cells. The first classifier C4.5 showed high accuracy (97.185%), precision (96.7%), recall (96.4%), and *F*-measure (96.7%). The overall correct rate of the Step 2 LR classifiers showed not only high accuracy (98.58%) and recognition rate of abnormal epithelial cells (98.6%), but also high areas under the ROC curve (0.996). The overall accuracy for the method we used was 95.805%, while the recognition rate for abnormal epithelial cells was 95.642%. Our method showed high accuracy and high abnormal epithelial cell recognition rate when compared with single classifier system and showed 2% higher abnormal cell recognition rate.

The cost of the system used in our study is estimated to be around 20,000$, including the equipment used to capture cell image such as industrial microscope (Olympus BX43) (~12,000$), high quality camera (IDS UI-3370CP-C-HQ) (~2000$), slides and staining materials (~500$), and software development and maintenance (~5500$). We think it is affordable for developing countries that are limited in budget and conditions. The application of this system will facilitate cervical cancer screening and prevention in developing countries and shorten the distance between developing countries and developed countries.

We will pay more attention to the larger samples in the future studies to confirm our promising findings.

## Figures and Tables

**Table 1:** Example morphologic and texture features of the nuclei of different type of cells.

	Step 1 classification				Step 2 classification	
Cell type	Epithelial cells	Lymphoid cells	Neutrophils cells	Junk cells	Normal epithelial cells	Abnormal epithelial cells
Cell image	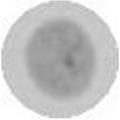	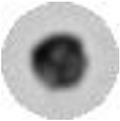	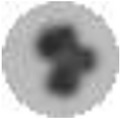	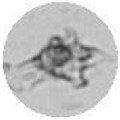	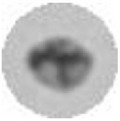	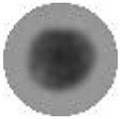
Area (μm^2^)	1071	403	342	972	538	2455
Circularity	0.843519	0.916002	0.765056	0.300962	0.753142	0.997429
Distance	17.397	10.2599	9.30611	15.8119	12.0604	26.8547
Sigma	1.16761	0.546327	1.61327	5.60155	1.2382	0.346338
Roundness	0.932884	0.946751	0.826644	0.645737	0.897334	0.987103
Sides	5.05552	5.63962	3.22911	2.30386	4.1358	11.0196
Mean	158.233	79.5434	100.567	128.506	117.796	123.245
Deviation	6.99297	41.8068	39.1483	28.4216	29.373	37.8975
Energy	0.0744681	0.00502435	0.00584795	0.0033976	0.00580941	0.0416018
Correlation	0.872788	0.891784	0.901872	0.852879	0.937312	0.93632
Homogeneity	0.716823	0.301341	0.281612	0.258799	0.368374	0.574155
Contrast	0.868347	25.5558	19.7018	15.9352	7.09665	12.4264
Convexity	0.97541	0.975787	0.914439	0.675939	0.967626	0.983574
*M*_11_	5434.79	−84.2779	1278.93	952.484	1756.51	−601.93
*M*_20_	105447	13084.7	12303.9	72230.5	18714	480713
*M*_02_	79455.2	12887.3	7861.95	131805	28798.7	478728
*I*_a_	106538	13115.8	12645.8	131821	29095.8	480881
*I*_*b*_	78364.5	12856.2	7520.04	72215.2	18416.8	478559
Entropy	4.58423	6.75867	6.62043	6.56861	6.61984	5.98287
Anisotropy	−0.549074	−0.484435	−0.478508	−0.533538	−0.523816	−0.640747
Compactness	1.1056	1.04565	1.30927	5.38011	1.11106	1.10762
ContLength (*μ*m)	121.983	72.7696	75.0122	256.35	86.669	184.853
Diameter (*μ*m)	39.3954	23.2594	23.7697	57.8705	29.4279	55.9464
Radius (*μ*m)	16.5	10.5	7	9	11.5	27
Rectangularity	0.809384	0.826425	0.760234	0.579734	0.77907	0.807594
Anisometry	1.16598	1.01004	1.29677	1.35107	1.25692	1.00242
Bulkiness	1.00102	1.00474	1.04771	1.29773	1.00501	1.00022
Structure-Factor	0.16717	0.0148302	0.358639	0.753318	0.263212	0.002.63965

**Table 2:** The accuracy of the four types of cells and the precision, the recall rate, and the *F*-measure of epithelial cells, from Step 1 classification.

Type	Assessment	NB	C4.5	*P* value
4 types of cells	Accuracy	92.065% (18413/20000)	97.185% (19237/20000)	0.0001

Epithelial cells	Precision	88.4% (4648/5256)	97% (4819/4970)	0.0001
	Recall rate	93.0% (4648/5000)	96.4% (4819/5000)	0.0001
	*F*-measure	90.6%	96.7%	

**Table 3:** The result of Step 2 classification. The accuracy and the precision as analyzed by NB and LR classifiers are shown.

Assessment	NB	LR	*P* values
Accuracy of 2 types of cells	84.1% (4205/5000)	98.58% (4929/5000)	0.0001

Precision of abnormal epithelial cells	88.3% (2107/2386)	98.6% (2455/2491)	0.0001
